# In-Syringe Micro Solid-Phase Extraction Method for the Separation and Preconcentration of Parabens in Environmental Water Samples

**DOI:** 10.3390/molecules23061450

**Published:** 2018-06-14

**Authors:** Geaneth Pertunia Mashile, Anele Mpupa, Philiswa Nosizo Nomngongo

**Affiliations:** Department of Applied Chemistry, University of Johannesburg, Doornfontein Campus, P.O. Box 17011, Johannesburg 2028, South Africa; petmashile2009@hotmail.com (G.P.M.); anelempupa@yahoo.com (A.M.)

**Keywords:** in-syringe micro solid-phase extraction, personal care products, response surface methodology, parabens, wastewater

## Abstract

In this study, a simple, rapid and effective in-syringe micro-solid phase extraction (MSPE) method was developed for the separation and preconcetration of parabens (methyl, ethyl, propyl and butyl paraben) in environmental water samples. The parabens were determined and quantified using high performance liquid chromatography and a photo diode array detector (HPLC-PDA). Chitosan-coated activated carbon (CAC) was used as the sorbent in the in-syringe MSPE device. A response surface methodology based on central composite design was used for the optimization of factors (eluent solvent type, eluent volume, number of elution cycles, sample volume, sample pH) affecting the extraction efficiency of the preconcentration procedure. The adsorbent used displayed excellent absorption performance and the adsorption capacity ranged from 227–256 mg g^−1^. Under the optimal conditions the dynamic linear ranges for the parabens were between 0.04 and 380 µg L^−1^. The limits of detection and quantification ranged from 6–15 ng L^−1^ and 20–50 ng L^−1^, respectively. The intraday (repeatability) and interday (reproducibility) precisions expressed as relative standard deviations (%RSD) were below 5%. Furthermore, the in-syringe MSPE/HPLC procedure was validated using spiked wastewater and tap water samples and the recoveries ranged between from 96.7 to 107%. In conclusion, CAC based in-syringe MSPE method demonstrated great potential for preconcentration of parabens in complex environmental water.

## 1. Introduction

Over the years living standards have improved and so has the production and use of cosmetics. Cosmetics are defined as any substance or mixture intended for use on the external parts of the human body, teeth and mucous membrane as defined by the European Union 1223/2009 (Article 2,1.Aa) [1]. This mainly includes personal care products (lotions, soup, perfume, deodorant), beauty products (nail polish, mascara, lip stick, etc.), hair care products (shampoo, hair dyes and gel, among others), oral products (mouthwash, toothpaste) [2]. The compounds or chemical substances within the cosmetics are connected to their intended use, that is, antioxidants, preservatives, fragrances, pigment, antimicrobial and UV-filters [3]. One of the most commonly used compounds in cosmetics, pharmaceuticals and personal care products (PPCPs) are parabens. These compounds are used primarily for their antimicrobial and antibacterial properties [4]. In cosmetics and personal care products the most used parabens are methyl, ethyl, propyl as well as butyl paraben which are often times used in combination with other preservatives [5]. They are preferred over other alternatives due to their low cost, broad spectrum of activity, thermal stability and applicability over a wide pH range [6].

Traditionally, parabens have been considered as low toxicity compounds, however more recent studies have found that certain parabens exhibit endocrine disrupting effects, which in turn can lead to a potential increase in breast cancer incidence [7] or the development of malignant melanomas [8], amongst other effects. This is compounded by the ability of parabens to be absorbed into the human skin due to dermal exposure to products which contain these compounds [4]. Moreover, parabens contain phenolic hydroxyl groups, which are capable of producing chlorinated degradation by-products when in contact with chlorinated water as in tap and swimming pool water [9]. The chlorinated by-products which originate from personal care products are said to be more toxic to aquatic organisms that their corresponding parent compounds [10]. The wide use of products containing such compounds has contributed to the direct introduction of parabens into the aquatic environment via the domestic and industrial wastewater route [11]. As a result this has prompted the development of various analytical methods for the determination of parabens in environmental samples (such as water, sewage influents and effluents) [12], soil [13,14], among others.

Over the years various analytical techniques for the determination of parabens in different matrices have been developed, including gas-chromatography (GC) [9,15,16], high performance liquid chromatography (HPLC) [13,17], capillary zone electrophoresis (CZE) [4] and ultra performance liquid chromatography [18,19] amongst others. HPLC methods are the most widely used for the analysis and determination of parabens since techniques such as GC require prior derivatization of the compounds [20].

These analytical techniques are often combined with pretreatment procedures to eliminate non-polar matrix components and also improve the selectivity, reliability and accuracy of the analysis [21,22]. As a result, various extraction and preconcentration techniques have to be used for analysis of parabens such as magnetic solid phase extraction (MSPE) [23], solid phase extraction (SPE) [19]; dispersive liquid liquid microextraction (DLLME) [24], stir-bar sorptive extraction (SBSE) [25] and solid phase microextraction (SPME) [26], amongst others.

Some conventional pretreatment procedures like LLE are scarcely used in the determination of parabens due to their tediousness and high consumption of solvents, so even when automated, the column length limits the ability to separate the different parabens [27]. Thus, conventional SPE has been used as a better alternative to solve these issues, which has resulted in an increase in the application of SPE for the determination of parabens [28]. SPE provides advantages such as preconcentration of trace analytes from larger sample volumes, reduction and elimination of matrix interferences, as well as less possibility of cross-contamination, simplicity, effectiveness of extraction, sensibility and high selectivity [20,22]. Moreover it can easily be automated with a wide variety of available sorbents which have been developed over the years [28]. However, SPE also presents some drawbacks such as limitations in treatment of large sample volumes, its time consuming nature, need for pre-treatment of sorbents prior to extraction, obstruction and clogging of cartridges and relatively low extraction efficiencies. In addition, aggregation [29] of particles may occur reducing the active surface area [30]. SPE cartridges used are usually made from plastics, which can absorb the analytes and hence increase the interference in the analysis [31]. Therefore, to eliminate the drawbacks of conventional SPE, sample pretreatment methods like solid phase microextraction (SMPE) were developed [32]. SPME was proposed as the better alternative as it allows the combination of sampling and sample extraction in a single step, which also eliminates the use of organic solvents for analysis of parabens. It is also a technique that is effective due to its advantages of high enrichment factor, sample operation and solvent-free microextraction [33].

Therefore, the aim of this work was to develop a quick, low solvent consumption, simple and easy to use analytical method based on in-syringe micro-solid phase extraction (MSPE) for the simultaneous preconcentration and determination of four common parabens in environmental matrices. A chitosan-coated activated carbon (CAC) composite was applied as a sorbent in SPME for the preconcentration of parabens in environmental water samples. Activated carbon was chosen due to its high adsorption capacity and affinity for organic compounds/pollutants [34]. This combination improves the performance and serves as a proper support for chitosan as an adsorbent by improving its mechanical and chemical properties. Moreover an added advantage is the lesser quantity of chitosan needed to create a new adsorbent without disrupting the adsorption capacity of the formed adsorbent significantly [35]. To the best of our knowledge, there are limited or no reports on the simultaneous extraction of parabens using a chitosan-coated activated carbon-based in-syringe MSPE method. The efficiency of the preconcentration method for extraction of parabens is due to the unique properties of the adsorbent. A response surface methodology (RSM)-based central composite design (CCD) was used to determine the optimum experimental conditions.

## 2. Results and Discussion

### 2.1. Characterization of Chitosan-Coated Activated Carbon (CAC)

The BET surface area of chitosan-coated activated carbon (CAC) is higher than that of activated carbon. An improvement on the properties of activated carbon was possible thanks to the presence of chitosan acting as a support for coating AC. A result of coating the chitosan is an increase in the surface area of the activated carbon while the pore volume and pore size are decreased due to the fact the chitosan can block the pores on the activated carbon, as seen in Table 1.

### 2.2. Optimization of in-Syringe SPME

**Figure 1:**
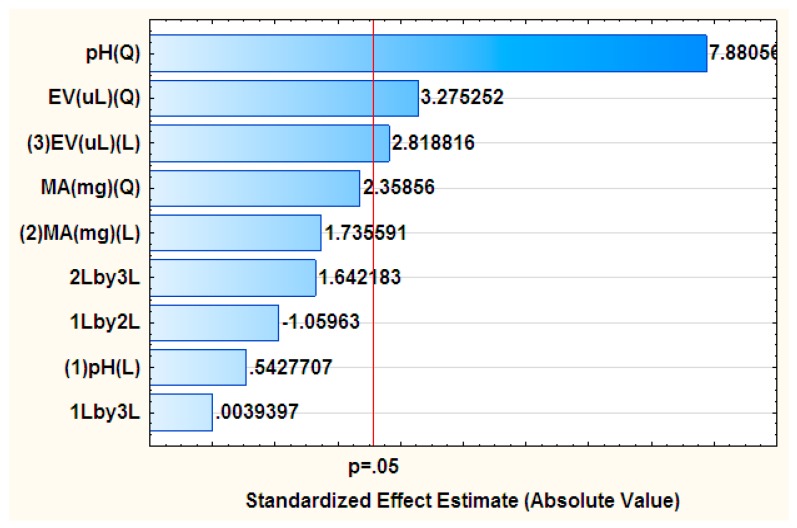
Pareto chart of standardized effects for variables in the preconcentration for parabens: The “Q” and “L” in brackets indicate whether the effects are quadratic or linear, respectively. The “2Lby3L” indicates the linear interactions between MA and EV, the “1Lby2L” referrers to the interactions between pH and MA and “1Lby3L” is for the the pH and EV interactions.

The optimization of factors affecting the experimental preconcentration procedure was carried out by means of an experimental design approach, whereby the effects of important variables such as mass of adsorbent, sample pH and eluent volume were determined using response surface methodology (RSM) based on a central composite design (CCD). The analysis of variance (ANOVA) represented in terms of Pareto chart was used to explore the significance of the effects on the in-syringe MSPE procedure (Figure 1). 

According to Figure 1 the effect of pH and mass of adsorbent were insignificant at the 95% confidence level, contrary to the eluent volume used for the preconcentration of parabens. The sample pH and all interactions were not significance at a 95% confidence level for the preconcentration of parabens. RSM was used to establish an extrapolative model which denotes changes in the response, depending on the contributing factors. The quadratic equation for the model illustrated the dependence of the analytical response (%recovery) with respect to the evaluated variables [36].

**Figure 2:**
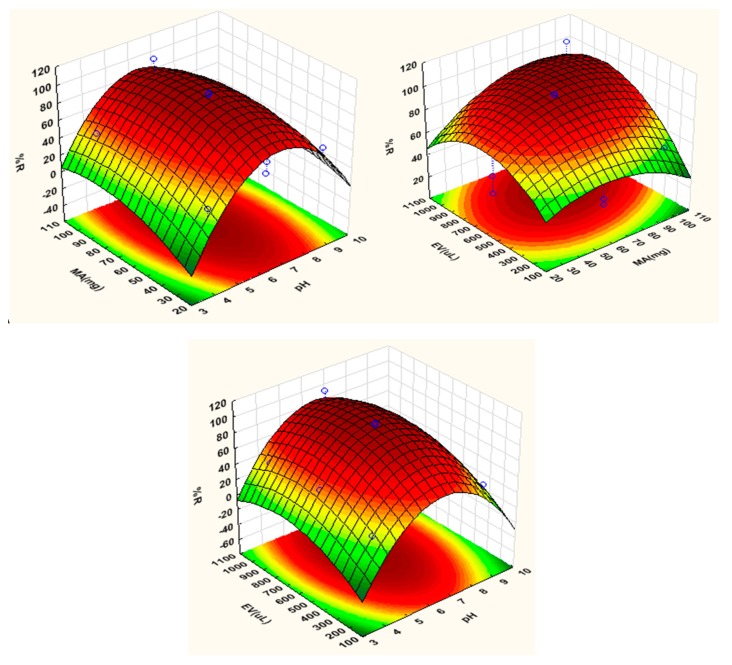
Response surface obtained for parabens after extraction and preconcentration by in-syringe MSPE.

The 3D response surface plots showing the analytical response against individual factors are shown in Figure 2, which illustrates the interaction of the pH with the mass of adsorbent and eluent volume as well as the interaction of eluent volume with mass of adsorbent, respectively. As seen an enhanced analytical response (%recovery) at pH values between 5 and 8 was observed. This demonstrated that the extraction and pre-concentration of parabens depended on the sample pH. In addition, it was observed that maximum recoveries were achieved at eluent volumes above 500 µL. This is because volumes below 500 µL were not enough to elute all the analytes from the adsorbent. The percentage recovery dependency as a function of the interaction between mass of adsorbent and pH revealed that the analytical response increased with increasing mass of adsorbent. This was probably due to the high surface area and small particle size of the chitosan-coated activated carbon.

**Figure 3:**
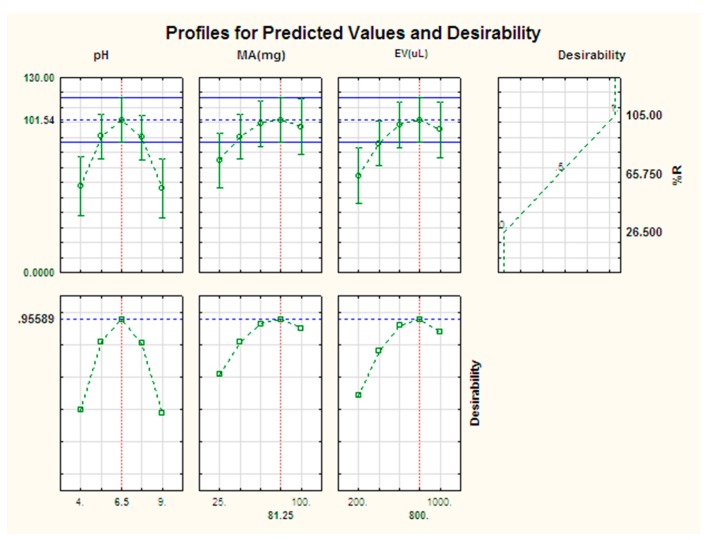
Profiles for predicated values and desirability function for pre-concentration of parabens.

In addition to the 3D response surface plots, the estimation of the optimum conditions was further carried out using the profile for predicted values and desirability. As seen in Figure 3 the desirability of 1.0 was assigned for maximum recoveries (105%), 0.0 for minimum (26.5%) and 0.5 for middle (65.8%). Figure 3 also shows the individual desirability scores for preconcentration of parabens (left hand side (bottom) and the, the desirability value of 1.0 was chosen as the target value to be used to obtain optimum conditions and the percent recovery obtained from plots for each parameter in the model is shown at the top left hand side. According to Khodadoust et al. [37], the figures on the top left hand side illustrate the changes in the level of each individual factor and its analytical response as well as its overall desirability. Therefore, based on the calculations from the 3D plots and desirability score of 1.0, the percentage recovery of parameters was optimized at 101.5 and the optimum conditions were mass of adsorbent 81 mg, eluent volume 800 and sample pH 6.5.

### 2.3. Adsorption Capacity of CAC

Under optimum conditions, the adsorption capacities of the CAC toward the parabens were investigated. To evaluate the adsorption capacity, 100 mg L^−1^ of paraben mixture solution was sonicated for 100 min at room temperature and the concentration at equilibrium were determined using HPLC-PDA. The adsorption capacities for each analyte are presented in Table 2.

## 3. Analytical Figures of Merit

Under optimal conditions the analytical performance of the proposed method was investigated in terms of precision (intra- and interday), linearity, limits of detection (LOD) and limits of quantification (LOQ) (Table 3). Seven point (triplicate) calibration curves for the parabens were constructed by plotting the peak area of the signal acquired using HPLC/PDA as a function of concentrations of parabens. 

Calibration curve linearity was observed in the range of 0.05–375 µg L^−1^ for methylP, 0.04–350 µg L^−1^ for ethylP, 0.04–380 µg L^−1^ for propylP and butylP 0.06–380 µg L^−1^. The correlation coefficient (*R*^2^) ranged from 0.9989 to 0.9995 (Table 4). The LODs, LOQs, enrichment factor and pre-concentration factor ranged 6–15 ng L^−1^, 20–50 ng L^−1^, 100 and 150–175, respectively.

The intraday precision (repeatability) was evaluated by analyzing fifteen successive replicates 50 µg L^−1^. The precision expressed in the form of relative standard deviation (%RSD) ranged from 1.5 to 2.1%. In addition, the interday precision (reproducibility of in-syringe device, triplicates, five working days) were between 2.0% and 3.9%.

Due to the absence of certified reference materials (CRM) for parabens in water samples, spike recovery experiments were performed for assessing the trueness of the developed method. The trueness validation procedure was carried out by spiking tap water at three concentration levels, that is low (10 ng L^−1^), middle (30 ng L^−1^), and high (50 ng L^−1^). Samples were analyzed in triplicate over a period of five days and the trueness of the method was assessed using relative bias and recovery (Table 4). As seen from Table 4, the relative bias varied between −2.7% and 1.7% while the recoveries were greater than 95% for all the studied analytes. In addition, the inter-day precision was less than 5% and these demonstrated the trueness of the developed method.

The analytical performance of the proposed in-syringe MSPE method for preconcentration and determination of parabens was compared with other different analytical procedures reported in the literature (Table 5). Thus, it can be observed that the proposed method has better analytical figures of merit compared to those reported by [19,38,39,40,41,42,43,44]. In addition, the analytical performances were comparable to those reported by [41] and lower than those reported by [42]. The results obtained in this work show that the in-syringe SPME-HPLC-DAD is efficient and a simple technique. Its optimum performances can be attributed to the attractive properties of the adsorbent. Thus, the method proved to have higher adsorption capacity, simple, cost effective and sensitive and efficient for the preconcentration and determination of parabens in water samples.

### 3.1. Analysis of Real Samples

The proposed method was also used in the preconcentration and determination of parabens in three kinds of real water samples (influent, effluent and tap water) that were analysed by HPLC-DAD after the in-syringe-SPME procedure. Under optimized conditions, the influent wastewater samples were spiked at two different levels (20 and 50 ng L^−1^, *n* = 3) and analyzed according to the proposed procedure. At first the sample was analyzed without spiking. This was done in order to evaluate the levels on parabens in the original sample and the results of the analysis are shown in Table 6. 

**Figure 4:**
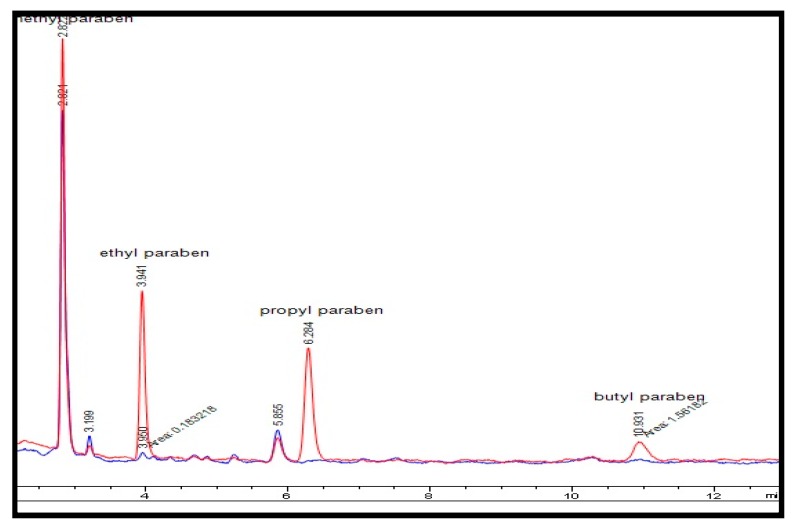
Chromatogram related to the extraction of target analytes (blue) wastewater (red) spiked wastewater at concentration level of 150 µg·L^−1^ of analytes.

Figure 4 present the chromatogram of unspiked effluent water and a spiked sample after both were preconcentrated using in-syringe MSPE. 

The percentage recoveries of paraben spiked at two levels were in the range of 97.5–99.4% with RSDs less than 5% (Table 7). The results demonstrated that the developed method can effectively preconcentrate and sensitively detect of trace amount of parabens in the presence of sample matrices.

The in-syringe MSPE/HPLC-DAD method was successfully applied for preconcentration and determination of parabens in real samples and the analytical results are presented in Table 6. The results obtained by the current method were not significantly different from those obtained by reference method. These results obtained indicated the applicability of the in-syringe MSPE/ HPLC-DAD method for determination of parabens in environmental water samples

## 4. Material and Methods

### 4.1. Reagents and Standards

Methyl-, ethyl-, propyl- and butyl parabens were purchased from Sigma-Aldrich (St. Loius, MO, USA). HPLC grade solvents, including methanol and acetonitrile, were from Sigma-Aldrich (St. Louis, MO, USA). Stock solutions of parabens (10 mg L^−1^) were prepared in ultra-pure water (Direct-Q^®^ 3UV-R purifier system, Millipore, Merck, Germany) and stored in a freezer at 4 °C. The working standards were prepared by subsequent dilution of stocks.

### 4.2. Instrumentation

Chromatographic analysis was carried out by an Agilent HPLC 1200 infinity series system, equipped with photodiode array detector (Agilent Technologies, Waldbronn Germany). The chromatograms were recorded at 250 nm and 260 nm. An Agilent Zorbax Eclipse Plus C18 column (3.5 µm × 150 mm × 4.6 mm) (Agilent, Newport, CA, USA) was operated at an oven temperature of 25 °C. The mobile phase was a mixture of 30% water (Mobile phase A) and 70% methanol (mobile phase C). A flow rate of 1.00 mL min^−1^ was used throughout the analysis. An OHAUS starter 2100 pH meter (Pine Brook, NJ, USA) was used for pH adjustments of the reagents and to measure the pH of samples.

The characterization of the adsorbents is crucial in their application for adsorption processes. The surface area (S_BET_) and pore size distribution of the adsorbent was determined from N_2_ adsorption-desorption isotherm using Brunauer, Emmett and Teller (BET) multipoint method using Surface Area and Porosity Analyzer (ASAP2020 V3. 00H, Micromeritics Instrument Corporation, Norcross, GA, USA). The pore volumes were calculated by the Barrerr-Joyner-Halenda method.

### 4.3. Methods

#### 4.3.1. Samples and Sample Collection

Both raw (influent) and treated (effluent) wastewater samples were used in this study. Sewage wastewater samples were collected in different points in the Daspoort wastewater treatment plant (Pretoria, Gauteng, South Africa). The samples were collected in pre-cleaned 500 mL glass bottles. After sampling the water samples were stored at 4 °C for a maximum of 1 week until being analysed.

#### 4.3.2. Preparation of CAC Adsorbent and Self-Made in-Syringe MSPE Device

Chitosan coated activated carbon adsorbent was synthesized according to the method described by Shariffard and colleagues [40]. Briefly, 5 g of activated carbon was treated with 0.2 mol L^−1^ oxalic acid for 4 h. The activated carbon was then washed with deionised water after filtration and dried in an oven at 70 °C for 12 h. After this, chitosan was also prepared by adding 5 g of chitosan to 500 mL in 0.2 mol L^−1^ oxalic acid solution under continuous stirring at 45–50 °C to form a viscous gel. Thereafter, the 5 g of the acid treated activated carbon was added slowly to the chitosan gel and stirred for 2 h at 45–50 °C. Then the activated carbon coated with chitosan beads were prepared by dropwise addition of the activated carbon gel into 0.7 M NaOH precipitation bath. The beads were filtered from NaOH bath and washed several times with deionised water to a neutral pH and dried in an air oven at 50 °C. The in-syringe SPE device [33] was prepared through slurry-packing CAC powders in the syringe-cartridge of with upper and lower filters. To discuss the methodology in detail, 81 mg CAC was added into 1 mL of deionized water and ultrasonicated to obtain a homogeneous dispersion. Afterwards, the dispersion was transferred to the barrel, and the plunger was pushed slowly to remove the deionized water from dispersion. During this process, CAC particles were gradually deposited on the surface of lower filter. The upper filter was subsequently embedded and compacted tightly to obtain a stable packing bed.

#### 4.3.3. In-Syringe SPE Procedure

For pre-concentration of parabens in model and real water samples, the self-made in-syringe-SPE device was first preconditioned with 1 mL methanol and 1 mL deionized water, respectively. After the conditioning step, the synthetic sample solution (100 µg L^−1^) was passed through the device packed with adsorbent and then discharged from syringe cartridge though pushing the plunger. The estimated residence time of the sample solution in MSPE device was about 1 min. Subsequently, the plunger was drawn-pushed for several times in the air to remove the residual water from the device. The adsorbent was washed with 1 mL 5% methanol/water to remove the interferences. Finally, the parabens adsorbed on CAC were eluted with 800 µL methanol by aspirating and dispensing the eluent for two cycles.

The eluate was injected into HPLC-DAD system for qualitative and quantitative analysis. After each extraction, the adsorbent was successively washed with 1 mL methanol and 1 mL water for the next extraction. The optimization of extraction and pre-concentration conditions was performed using experimental design approach based central composite design (CCD). The factors investigated included mass of adsorbent, pH of sample solution and eluent volume (Table 8). The statistical analysis was achieved using Statistica software (Version 13, StatSoft, Inc., Tulsa, OK, USA).

## 5. Conclusions

The study proposed a method which allowed for the simultaneous determination of trace levels of four common parabens in wastewater. Its procedure was based on an in-syringe-SPME pre-concentration technique followed by HPLC-DAD analysis of the parabens. This method was successfully applied to the analysis of wastewater collected from the Daspoort wastewater treatment plant (WWTP, Pretoria, Gauteng, South Africa). Compared to other methods reported in the literature, this technique offers simplicity, ease of use, cost effectiveness and lower consumption of organic solvents. It could effectively detect the most commonly used parabens (methyl and propyl paraben) throughout the study as the concentration of methyl paraben remained constant and propyl paraben was detected in all three analytes. Thus the adsorbent CAC was highly effective in the pre-concentration procedure for the analyses of the parabens

## Figures and Tables

**Table 1:** Characteristics of adsorbent.

Parameter	Activated Carbon	Chitosan Coated Activated Carbon
BET surface area (m^2^/g)	1075.45	1181
Pore Volume (cm^3^/g)	0.7553	0.733
Pore size (nm)	4.839	4.545

**Table 2:** Adsorption capacity (mg/g) of chitosan coated activated carbon.

Paraben	Adsoption Capacity (mg/g)
Methyl	227
Ethyl	236
Propyl	256
Butyl	241

**Table 3:** Analytical figures of merit of the proposed in-syringe MSPE/HPLC method for the preconcentration and determination of parabens.

Paraben	Linearity (µg L^−1^)	Correlation Coefficient (*R*^2^)	LOD (ng L^−1^)	LOQ (µg L^−1^)	Precision (%RSD)	
					Intraday	Interday
Methyl	0.05–375	0.9991	12	40	2.1	3.3
Ethyl	0.04–350	0.9989	10	33	1.8	2.5
Propyl	0.04–380	0.9995	6	20	1.6	3.9
Butyl	0.06–380	0.9992	15	50	1.5	2.0

**Table 4:** Trueness experiment using three level concentrations of each analyte.

Paraben	Added Value (ng L^−1^), *n* = 5	Measured Value (ng L^−1^), *n* = 5	%RSD	Trueness	
				%Recovery	%Relative Bias
Methyl	10	9.87 ± 0.17	1.7	98.7	−1.3
	30	30.5 ± 1.3	4.3	102	1.7
	50	49.8 ± 1.8	3.6	99.6	−0.4
Ethyl	10	9.92 ± 0.23	2.3	99.2	−0.8
	30	29.8 ± 0.5	1.7	99.3	−0.7
	50	48.9 ± 1.5	3.1	97.8	−2.2
Propyl	10	9.91 ± 0.12	1.2	99.1	−0.9
	30	29.2 ± 0.6	2.1	97.3	−2.7
	50	50.3 ± 1.3	2.6	101	0.6
Butyl	10	9.76 ± 0.16	1.6	97.6	−2.4
	30	29.7 ± 0.9	3.0	99.0	−1.0
	50	50.1 ± 1.2	2.4	100	0.2

**Table 5:** Comparison of the proposed microextraction method with literature for determination of parabans.

Parabens	Matrices	Analytical Method	LOD (ng L^−1^)	References
Methyl, ethyl, propyl, isopropyl, butyl, isobutyl, benzyl	Wastewater, River water	SPE/HPLC-CL	0.08–0.44	[42]
Methyl, ethyl, propyl	Cosmetics, beverages, water	HPLC-UV/ SUPRAS	70–500	[43]
Methyl, propyl, butyl, benzyl and benzophenone-4	Wastewater (Influent and effluent)	SPE-HPLC-MS/MS	0.8–4.8	
Methyl, ethyl, propyl, isopropyl, butyl, benzyl	Wastewater	SPE/UPLC-MS/MS	221–21,423	[19]
Methyl, ethyl, propyl, butyl	Wastewater, river water, swimming pool water	DLLME/GC-MS/MS	3.90–27.5	[40]
Methyl, ethyl, propyl	Toothpaste, mouth rinse, shampoo, tap water, river water	DLLME/HPLC-UV	5–20	[41]
Methyl, propyl	Underground water	HF-MMLLE/HPLC-DAD	500–4600	[39]
Ethyl, propyl, isobutyl, butyl	Lake and river water	HPLC-UV/DF-µLPME	1600–3500	[38]
Methyl, ethyl, propyl, butyl	Wastewater, tap water	In-syringe MSPE/HPLC-PDA	6–15	This work

Extraction methods: SPE: solid phase extraction, SBSE: stir-bar sorptive extraction, SPME: solid phase micro-extraction, DLLE; dispersive liquid-liquid extraction, MSPD: matrix solid-phase dispersion, DF-µLPME: double-flow microfluidic based liquid phase micro-extraction, HF-MMLLE: hollow fiber-microporous membrane liquid-liquid extraction. Analytical methods: HPLC-CL: high performance liquid chromatography-chemiluminescence, UPLC: ultra-performance liquid chromatography-mass spectrometry, GC-MS: gas chromatography-mass spectrometry.

**Table 6:** Analysis of parabens in real samples (concentration in ng L^−1^) using in-syringe MSPE/ HPLC-DAD, *n* = 6, degrees of freedom = 5.

Samples	Methyl P	Ethyl P	Propyl	Butyl
Influent 1	947 ± 3	168 ± 2	108 ± 1	<LOQ
Influent 2	889 ± 2	267 ± 3	1988 ± 9	133 ± 3
Effluent 1	392 ± 3	22.4 ± 0.9	<LOQ	<LOQ
Effluent 2	<LOQ	<LOQ	1396 ± 5	132 ± 2
Tap water	<LOQ	<LOQ	629 ± 3	<LOQ

**Table 7:** Validation of in-syringe MSPE for microextraction of parabens in spiked samples (*n* = 4).

Added (ng L^−1^)	Methyl		Ethyl		Propyl		Butyl	
	Found (ng L^−1^)	%Recovery	Found (ng L^−1^)	%Recovery	Found (ng L^−1^)	%Recovery	Found (ng L^−1^)	%Recovery
0	392 ± 3 ^a^	-	22.4 ± 0.9		ND		ND	-
20	412 ± 5	98.0 ± 1.2	42.2 ± 1.2	99.0	19.5 ± 0.5	97.5 ± 2.6	19.6 ± 0.4	98.0 ± 2.0
50	442 ± 3	99.5 ± 0.8	71.6	98.4	49.6 ± 2.1	99.2 ± 4.2	49.7 ± 0.5	99.4 ± 4.0

^a^ mean ± standard deviation (*n* = 3).

**Table 8:** Factors and levels of experimental design.

Variables	Low Level (−1)	Centre Point (0)	High Level (+)
Mass of Adsorbent (mg)	25	72	100
Sample pH	4.0	6.6	9.0
Eluent Volume (µL)	200	720	1000
